# Methylation Defect in Imprinted Genes Detected in Patients with an Albright's Hereditary Osteodystrophy Like Phenotype and Platelet Gs Hypofunction

**DOI:** 10.1371/journal.pone.0038579

**Published:** 2012-06-05

**Authors:** Benedetta Izzi, Inge Francois, Veerle Labarque, Chantal Thys, Christine Wittevrongel, Koen Devriendt, Eric Legius, Annick Van den Bruel, Marc D'Hooghe, Diether Lambrechts, Francis de Zegher, Chris Van Geet, Kathleen Freson

**Affiliations:** 1 Center for Molecular and Vascular Biology, University of Leuven, Leuven, Belgium; 2 Departement of Pediatrics, University of Leuven, Leuven, Belgium; 3 Center for Human Genetics, University of Leuven, Leuven, Belgium; 4 General Hospital Sint Jan Brugge, Brugge, Belgium; 5 Vesalius Research Center, University of Leuven and VIB, Leuven, Belgium; University of Bonn, Institut of experimental hematology and transfusion medicine, Germany

## Abstract

**Background:**

Pseudohypoparathyroidism (PHP) indicates a group of heterogeneous disorders whose common feature is represented by impaired signaling of hormones that activate Gsalpha, encoded by the imprinted *GNAS* gene. PHP-Ib patients have isolated Parathormone (PTH) resistance and *GNAS* epigenetic defects while PHP-Ia cases present with hormone resistance and characteristic features jointly termed as Albright's Hereditary Osteodystrophy (AHO) due to maternally inherited *GNAS* mutations or similar epigenetic defects as found for PHP-Ib. Pseudopseudohypoparathyroidism (PPHP) patients with an AHO phenotype and no hormone resistance and progressive osseous heteroplasia (POH) cases have inactivating paternally inherited *GNAS* mutations.

**Methodology/Principal Findings:**

We here describe 17 subjects with an AHO-like phenotype that could be compatible with having PPHP but none of them carried Gsalpha mutations. Functional platelet studies however showed an obvious Gs hypofunction in the 13 patients that were available for testing. Methylation for the three differentially methylated *GNAS* regions was quantified via the Sequenom EpiTYPER. Patients showed significant hypermethylation of the XL amplicon compared to controls (36±3 *vs.* 29±3%; p<0.001); a pattern that is reversed to XL hypomethylation found in PHPIb. Interestingly, XL hypermethylation was associated with reduced XLalphaS protein levels in the patients' platelets. Methylation for NESP and ExonA/B was significantly different for some but not all patients, though most patients have site-specific CpG methylation abnormalities in these amplicons. Since some AHO features are present in other imprinting disorders, the methylation of *IGF2*, *H19*, *SNURF* and *GRB10* was quantified. Surprisingly, significant *IGF2* hypermethylation (20±10 *vs*. 14±7%; p<0.05) and *SNURF* hypomethylation (23±6 *vs*. 32±6%; p<0.001) was found in patients *vs*. controls, while *H19* and *GRB10* methylation was normal.

**Conclusion/Significance:**

In conclusion, this is the first report of methylation defects including GNAS in patients with an AHO-like phenotype without endocrinological abnormalities. Additional studies are still needed to correlate the methylation defect with the clinical phenotype.

## Introduction

Heterozygous inactivating mutations affecting the *GNAS* gene have been reported to cause Albright's Hereditary Osteodystrophy (AHO, MIM 300800), a complex and broad phenotype mostly characterized by short stature, obesity, round face, subcutaneous calcifications, brachydactyly and cognitive impairment [1]–[4]. Patients carrying *GNAS* loss-of-function mutations on maternally inherited alleles have pseudohypoparathyroidism type Ia (PHP-Ia, MIM 103580) that is characterized by AHO and resistance to multiple stimulatory G protein-coupled hormones (*e.g.* Parathormone (PTH) and others) [5]–[10], while patients with paternally inherited *GNAS* mutations are reported as having only AHO features or pseudopseudohypoparathyroidism (PPHP) (**Table 1**) [2], [4], [11], [12]. Progressive Osseous Heteroplasia (POH, MIM 166350) describes a severe disease characterized by ectopic bone formation that affects not only the subcutis, but also the skeletal muscle and the deep connective tissue. POH is considered as an extreme variant of PPHP that can be associated with some AHO features and is also caused by paternally inherited *GNAS* inactivating mutations (**Table 1**) [13]. *GNAS* imprinting defects have extensively been described in pseudohypoparathyroidism type Ib (PHP-Ib, MIM 623233) patients [11], [14] with hormone resistance to PTH and TSH only and having no AHO. However, recent studies have shown the presence of epigenetic *GNAS* defects in PHP-Ia patients without mutations in the *GNAS* coding region (**Table 1**) [15]–[19]. These findings suggest a reclassification of PHP-Ia and PHP-Ib patients as extreme ends of one heterogeneous group of *GNAS* (epi)genetic defects. The latter is further supported by the Gs functional overlapping between PHP-Ia and PHP-Ib recently reported, where Gsalpha hypofunction, determined either in isolated erythrocyte membranes or in platelets, has been detected also in patients with GNAS imprinting mutations, AHO features and hormone resistance [15], [20], [21]. Gsalpha loss of function is also a finding in PPHP patients [20], [22], [23]. However, despite the fact that large-scale studies showed an association between AHO phenotype and loss of Gs activity [22], [24]–[26], only a small number of PPHP subjects have inactivating *GNAS* mutations. The severity of the AHO phenotype varies greatly between patients, and some patients have only few features of the syndrome.

**Table 1:** Phenotypic, Molecular Genetic and Platelet Gs protein activity in relation to *GNAS* pathology.

	PHP-Ib	PHP-Ia	PPHP	POH
**AHO features**	no	yes	yes	rarely
**PTH resistance**	yes	yes	no	no
**Heterotopic ossification**	no	no	no	yes
**GNAS defect**	Epigenetic GNAS defects	Mutations in exons 1-13/ Epigenetic GNAS defects	Mutations in exons 1-13	Mutations in exons 1–13
**Platelet Gsα activity^20^**	Mildly reduced	reduced	reduced	/
**Transmission**	maternal	maternal	paternal	paternal

Some clinical characteristics of AHO are also reported in imprinting syndromes Silver-Russell, Beckwith-Wiedemann, Prader-Willi and Angelman that are mainly characterized by defects in growth, behaviour and/or development. To further support the common soil of imprinting disorders, an ‘imprinting gene network’ that regulates embryonic growth and differentiation dependent on Zac-1 (also known as pleiomorphic adenoma gene-like 1 (PLAGL1)) regulation has been identified [27]. A subset of imprinting genes has been found to influence growth progression via coordination of the glucose-regulated metabolism [28]. Among those genes, together with *GNAS* also the *IGF2/H19* cluster and the *SNURF*/*SNRPN* regions have been described to play a causative role in embryonic growth defects. DNA methylation defects involving imprinting control region 1 (ICR1) of the *IGF2*/*H19* locus for which methylation abnormalities result in two growth disorders with opposite phenotypes: the overgrowth disorder Beckwith-Wiedemann syndrome [29] with maternal *H19*-ICR1 hypermethylation and the growth retardation disorder Silver–Russell syndrome [30] with paternal *H19*-ICR1 loss of methylation. Prader-Willi and Angelman syndromes [31] are distinct neurodevelomental disorders that are associated with the deletion of the chromosomal 15q11–13 region, loss of imprinting or uniparental disomy of chromosome 15. The *SNURF*/*SNRPN* region is hypermethylated in some Prader-Willi syndrome patients [31].

We here study the methylation of the growth regulatory imprinted genes *GNAS* (NESP, XL and ExonA/B amplicons), *IGF2*/*H19* and *SNURF* in 17 patients with some typical AHO features that mainly include in common growth retardation and brachydactyly. Methylation studies of GRB10 are also performed, as the imprinting of this gene is not actually linked to growth regulation but rather to behaviour [32]. All 13 patients that were available for platelet Gs testing showed a significant platelet Gs hypofunction but they did not carry *GNAS* coding mutations.

## Materials and Methods

### Ethics Statement

Verbal informed consent to collect blood samples for advanced non-routine diagnostic procedures was obtained from the participants and/or their legal representatives. This strategy is in agreement with the Belgian Law and local regulations and was specifically approved for this study by the Ethics Committee of the Katholieke Universiteit Leuven- University of Leuven. The Ethics Committee of the Katholieke Universiteit Leuven- University of Leuven, also waived the need for formal approval by the ethical review board.

### Participants

Patients enrolled in this study were followed at or referred to the pediatric endocrinology department of the University Hospital in Leuven (Belgium).

Patients were selected based on having AHO features, mostly with severe short stature, mental retardation or behavioural problems, clinodactyly or short metacarpals. Few patients also showed obesity and none of them presented with subcutaneous calcifications. One patient (patient 5) showed heterotopic ossifications, and was diagnosed with Progressive Osseous Heteroplasia [33], [34]. Other clinical characteristics were also present and are reported in **Table 2**. None of the patients had abnormal PTH, calcium or phosphate values.

### Functional platelet Gs pathway test

**Table 2:** Clinical patients' characteristics and platelet Gs activity.

Case	Birth weight	Height Z-score	Brachydactily (RX diagnosis)		other bone diseases	ID	SSC	Ob	RF	others	Ca, PTH and TSH levels	Gs test Prostin IC50 (ng/ml) 59,2±24,49 (n = 24)	Gs test Iloprost IC50 (ng/ml) 1,06±0,37 (n = 24)
1	3,1	−1,56	MC IV	MT V	enchondroma	YES	NO	NO	NO	cafè-au-lait spots	Normal	ND	ND
2	3,1	−1,5	MC IV, V	MT II, V	NO	NO	NO	NO	YES	-	Normal	ND	ND
3	NA	−0,73	MC V	-	clinodactyly	YES	NO	NO	YES	epilepsy, cryptorchidism, spastic paraparesis, strabism, café-au-lait spots,macrocephaly	Normal	ND	ND
4	2,45	−2,59	MC II, IV	MT I	broad thumbs, clinodactyly	YES°	NO	NO	YES	strabism, coarctatio aortae	Normal	ND	ND
5	adopted	−2,89	MC III, IV	MT III	synostosis, heterotopic calcifications	YES	NO	NO	YES	migraine, pubertas praecox	Normal	330**	>3**
6	2,03*	−3.6	MC IV	-	scoliosis	YES	NO	NO	NO	-	Normal	150**	2,**
7	3,24	−2.6	-	-	short fingers clinodactily, broad thumbs, red bone age	YES	NO	NO	NO	Pubertas praecox	Normal	122**	1,94**
8	2,96	−3.3	-	-	Retarded bone age,Frontal bossingTooth agenesis	NO	NO	NO	NO	-	Normal	367**	>3**
9	3,35	−3.2	-	-	broad hands	NO	NO	NO	NO	-	Normal	144**	2,06**
10	3,4	−2.3	-	-	non fusion of sacral vertebraeS1/S2, broad thumbs	NO	YES	NO	NO	-	Normal	433**	1,25**
11	1,96*	−2.6	-	-	clinodactily, retarded bone age	NO	NO	NO	NO	-	Normal	322**	>3**
12	3,64	−2.8	MC I, IV, V	MT I	osteochondroma	NO	NO	NO	NO	-	Normal	410**	1,95**
13	2,63*	−3.4	MC III uni, IV, V	MT I	exostose, osteochondroma,broad thumbs	YES	NO	NO	NO	synophris	Normal	250**	>2,5**
14	3,16	−3.2	MC IV, V	-	broad thumbs	YES	NO	NO	YES	synophris	Normal	>1000**	2,45**
15		−3.4	-	-	retarded bone age, broad hands	NO	NO	NO	NO		Normal	100**	1,94**
16	3,05	−1,16	MC IV, V	normal	NO	NO	NO	YES	YES	orchidopexy, coarctatio	Normal	125**	1.55**
17	3,95	0.52	normal	normal	Small and broad hands	YES	NO	YES	YES	eccchymoses, hirsutism, macrocephalie, praecox pubertas	Normal	305**	>2.5**

* Small-for-Gestational-Age (SGA); ∧Gilles de la Tourette; °ADHD; SSC: subcutaneous calcifications; Ob: obesity; RF: round face; ID: Intellectual disability; NA: not available.

** vs. crls, p<0.05 The concentration of Gsα agonist to inhibit the collagen-induced platelet aggregation by 50 % (IC_50_) is indicated between brackets for 24 normal controls. A Gsα hypofunction is defined as requiring a significantly higher IC_50_ value.

The platelet aggregation-inhibition test was performed as described [35]–[38]. Samples were processed within 3 hours after blood drawing. Different concentrations of a Gs agonist being prostaglandin E_1_ (PGE_1_, Prostin®; 0−1 μg/ml; Pfizer Inc., NY, USA) or the stable prostacyclin analogue Iloprost (Ilomedine® 0−5 ng/ml; Bayer Schering Pharma AG, Berlin, Germany) were added one minute prior to induction of aggregation with collagen (2 µg/ml). The 50% inhibitory concentration (IC_50_) was evaluated for each Gs agonist from the patient's response curve and compared to the mean IC_50_ measured on platelets of a group of controls (n = 24) for the same agonist [20].

### Genetic analysis of GNAS locus

DNA was extracted from leukocytes from all patients. Exons 1 to 13 of *GNAS* were amplified and sequenced using conditions previously described [20]. The presence of STX16 deletions was investigated as described [20], [39]–[41]. To rule out the presence of other deletions in the upstream *GNAS* region, we performed genotyping of different SNPs by PCR and direct sequencing within the NESP55, XL and Exon A/B regions (for overview of all SNPs see **Table S1**).

### GNAS, IGF2, H19, SNURF and GRB10 methylation analysis

Genomic DNA (1 ug) was used for bisulfite treatment with the MethylDetector™ bisulfite modification kit (Active Motif, Carlsbad CA) as described [19].

NESP, XL, Exon A/B and *SNURF* methylation was studied via Sequenom EpiTYPER technology using primers and conditions already reported [19]. New amplicons to study *IGF2*, *H19* (ICR1 region) and GRB10 regions were designed using the Sequenom EpiDesigner software. Primers and amplicons characteristics are reported in **Tables** **3** **and** **4**. All PCR amplifications were performed in triplicate. When the triplicate measurements had a SD equal to or greater than 0.10, all data for the sample involved were discarded (removing 8% of measurements). Sequenom peaks with reference intensity above 2, overlapping and duplicate units were excluded from the analysis.

**Table 3:** Primers used in the Sequenom study to amplify *IGF2*, *ICR1/H19* and *GRB10* regions.

primer's name	nucleotide sequence
**IGF2_F**	5′-aggaagagagGTTGGAGGGTTTTAAAGTGGGG-3
**IGF2_R**	5′-cagtaatacgactcactatagggagaaggct CAACTCAAATCCTACCTACATAA-3′
**H19_4_F**	5′-aggaagagagTAGTTTAAGTTTTTTTTGGATGGGG-3′
**H19_4_R**	5′-cagtaatacgactcactatagggagaaggct AAAACAACAATAACACTCCCAACTC-3′
**H19_14_F**	5′-aggaagagagTTTGGTAGGTTTAAGAGTTTAGGGG-3′
**H19_14_R**	5′-cagtaatacgactcactatagggagaaggct AAAACCCTACAAAAAAAATCTCACC-3′
**GRB10_F**	5′-aggaagagagGTTTAAATGGGATTTTATTTTGTTT-3′
**GRB10_R**	5′-cagtaatacgactcactatagggagaaggct AATCCCTAATTCTCATAACAACCCT-3′

**Table 4:** Chromosomal location of the *IGF2*, *H19* and *GRB10* amplicons used in the Sequenom study.

name amplicon	chromosome	start*	end*	size (bp)	theoretical number of CpGs per amplicon	effective number of GpCs studied via the Sequenom EpiTYPER
**IGF2**	11	2161350	2161846	496	45	30
**H19_4**	11	2021131	2021590	459	19	15
**H19_14**	11	2022413	2022822	409	17	10
**GRB10**	7	50850662	50851041	379	20	18

* Nucleotide positions according to the February 2009 human reference sequence (GRCh37/hg19) produced by the International Human Genome Sequencing Consortium.

The sequence and chromosomal location of all amplicons are shown in **Figures S1, S2, S3.**

### Genetic analysis of IGF2 and SNURF amplicons

To rule out the presence of SNPs that could interfere with the methylation detection sensitivity in the *IGF2* and *SNURF* amplicons, we have screened for the presence of SNPs in the same region that was used for the Sequenom analysis and its surrounding region. A list of all the *IGF2* and *SNURF* SNPs are reported in **TablesS2** **and S3** that could exclude also deletions in the loci as most patients are heterozygous for the intronic SNPs rs734351 and rs2855523.

### Platelet immunoblot analysis

Platelet immunoblot analysis for XLalphas, Gsalphas and CAP-1 was performed as described [20] Platelets isolated from citrated blood were directly lysed in ice-cold PBS containing 1% igepal CA-630 (Sigma Chemical, St. Louis, MO), 2 mmol/liter Na_3_VO_4_, 1 mmol/liter EDTA, 1 mmol/liter phenylmethylsulfonyl fluoride, 2 mmol/liter dithioerythreitol, 1% aprotinin, and 2 mmol/liter NaF, and incubated on ice for 60 min. Platelet extracts (50 μg) were mixed with Laemmli sample buffer and resolved by SDS/PAGE. Blots were revealed with a monoclonal anti-Gsα antibody [42] a monoclonal anti-XLαs antibody (11F7) [43] or a monoclonal anti-CAP1 antibody as loading control (Santa Cruz Biotechnology Inc.). Bands were quantified using the Java image processing program ImageJ 1.34 g (NIH Image software).

### Statistical analysis

Average of CpGs methylation for each amplicon was calculated for both controls and patients samples. Statistical analysis was performed using PRISM 5.0a software. Two-tailed unpaired T-test (p<0.05) was used to study group methylation differences between PPHP patients and healthy controls for all the imprinting control regions studied and to evaluate protein expression differences.

A more individual statistical approach was then performed comparing each patient's Sequenom CpG value or amplicon average with the distribution of values of the same variable measured in a group of healthy controls (n = 41 for NESP, n = 48 for XL and *GRB10*, n = 47 for Exon A/B, n = 45 for *IGF2*, n = 33 for *H19*, n = 35 for *SNURF*) (Z-test, P<0.05). Values with a Z-score ≤−2 and ≥+2 were considered significantly hypo- or hypermethylated, respectively. Normality test was assessed with SPSS 12.5 software to study the control population values distribution.

## Results

### Platelet Gs function

We studied platelet Gs activity in 13 PPHP patients with variable AHO features as reported in **Table** **2**. For patients 1 to 4 platelet testing could not be performed since only a DNA sample was available for further analysis. When platelet aggregation was induced with collagen in the patients, after preincubation with either prostaglandin E1 (Prostin) or a stable prostacyclin analogue (Iloprost), significantly higher concentrations of both Gs agonists were required to achieve the 50% inhibition of platelet aggregation (IC_50_), as compared to the healthy controls. This platelet aggregation-inhibition Gs test was performed in 24 healthy controls and we compared their mean IC_50_ values for patients.

### Genetic analysis of GNAS

Since our patients with an AHO-like phenotype were clinically diagnosed as having PPHP or POH (only for patient 5) and had platelet Gs hypofunction, *GNAS* screening for inactivation mutations was performed using leukocyte gDNA for sequencing the PCR amplified 13 exons, including exon/intron boundaries. No *GNAS* coding mutations were found in any of the patients. All patients were heterozygous for at least one of the studied *GNAS* region SNPs, excluding small chromosomal deletions within the *GNAS* cluster (**Table** **S1**). In addition, patients 5 to 17 were previously studied for copy number variants within the GNAS locus or its surrounding region and found to be negative [44].

### Study of GNAS methylation

**Figure 1:**
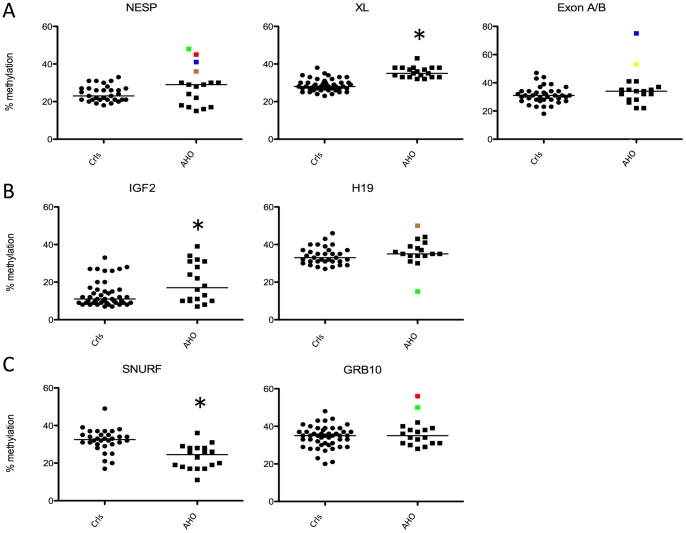
Overall *GNAS*, *IGF2*, *H19*, *SNURF* and *GRB10* methylation in AHO-like patients. Dot plot representation of overall methylation values (averages expressed as % of methylation) for NESP, XL, Exon A/B (A), *IGF2*, *H19* (B), *SNURF* and *GRB10* (C) in AHO-like patients (indicated as ‘PPHP’) vs. the control population (indicated as ‘crls’). Individuals with significant hyper- or hypomethylation (patients 1 to 5) in the NESP, Exon A/B, H19 and GRB10 are indicated as follow: patient 1 =  red, 2 =  green, 3 =  blue, 4 =  brown, 5 =  yellow. Medians are displayed as black lines. ** p<0.01 and * p<0.05, two-tailed unpaired T-test.

**Figure 2:**
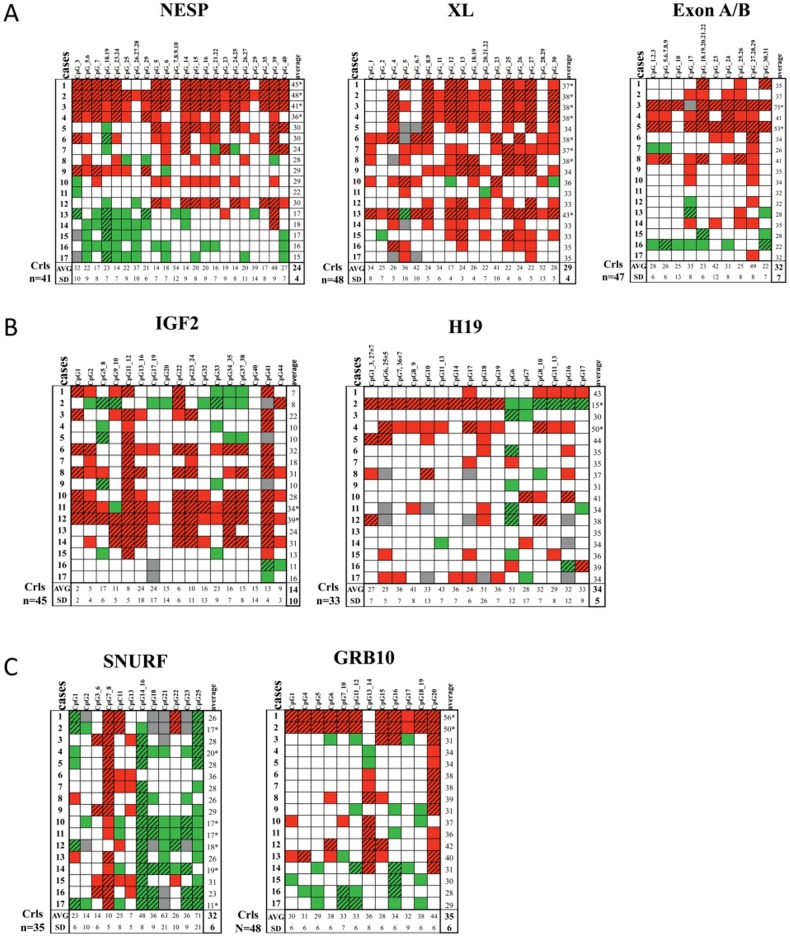
*GNAS*, *IGF2*, *H19*, *SNURF* and *GRB10* methylation at single CpG sites for AHO-like patients. Single CpG site methylation values rapresentations for all patients studied via Sequenom EpiTYPER mass-array for NESP, XL, exon A/B (A), *IGF2*, *H19* (B), *SNURF* and *GRB10* (C) amplicons. % of methylation are reported as mean of three replicates from at least two separate plates and two independent DNA bisulphite treatment. White include the normal methylation values that are within the mean +/− SD value of the indicated number of normal controls. Values that are significantly hyper- or hypomethylated are depicted as red or green diagonal striped rectangles, respectively (Z-test, p<0.05). Red or green rectangles indicate methylation values that are outside the SD values but are not yet significant, indicative for a trend towards hyper or hypomethylation, respectively. Grey rectangles are CpG values that failed in the analysis. The mean (AVG) and Standard Deviation (SD) for each CpG in the controls are shown in the last rows. The last column in white shows the overall degree of methylation for the complete amplicon for each patient and the mean and SD for the controls. * Z-test, p<0.05.

*GNAS* methylation was screened for the three amplicons NESP, XL and ExonA/B using the Sequenom EpiTYPER as we previously optimized for PHP-Ib and PHP-Ia cases [19]. We could observe a significant hypermethylation for the XL amplicon in patients *vs*. controls (36±3 vs. 29±3% (mean±SD); T-test, p<0.001; **Figure** **1A**). Interestingly, this is the opposite pattern of the methylation defect described for PHP-Ib and PHP-Ia patients having pronounced XL hypomethylation [19]. Overall methylation that includes all studied CpGs in the amplicons for NESP and ExonA/B did not show any significant difference between patients and controls (**Figure** **1A**) though some separate patients (patient 1, 2 and 3 for NESP and patient 3 and 5 for ExonA/B) showed a significant difference in overall methylation (**Figure** **2A**). However, the study of single CpGs within these amplicons showed significant hyper- (red) or hypo- (green) methylation (Z-test, p<0.05) for both the NESP and ExonA/B amplicons and for almost all patients (**Figure** **2A**). Based on the analysis of the single CpGs in NESP and ExonA/B (not for XL), some patients seemed to cluster in subgroups but these clusters did not correlate further with the clinical severity of AHO phenotype.

### Study of XLalphaS and Gsalpha expression in platelets

**Figure 3:**
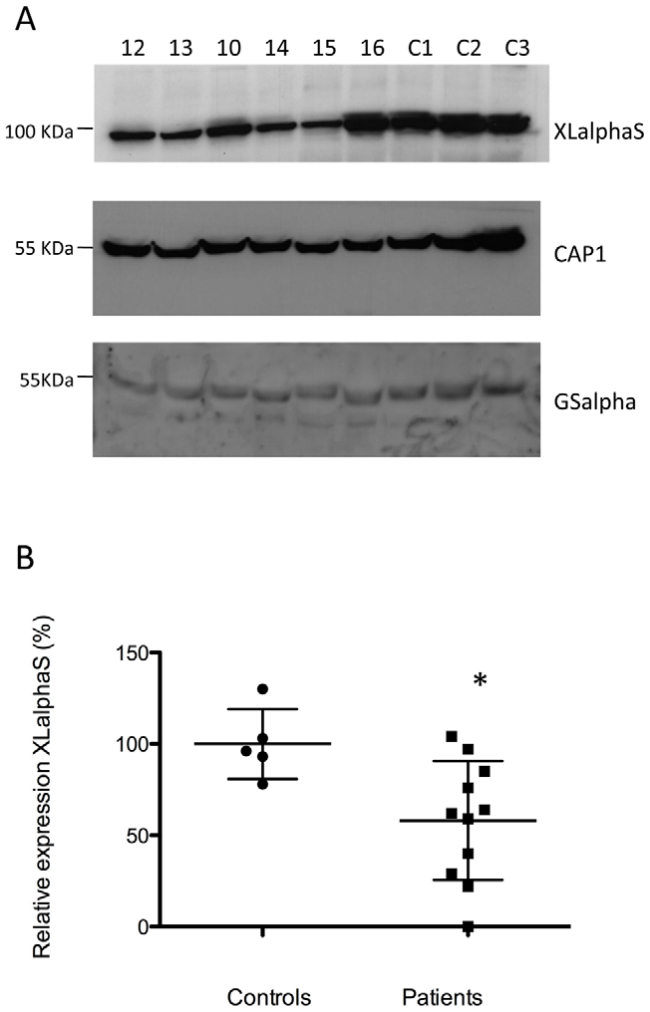
XLalphaS and Gsalpha expression in platelets from AHO-like patients. XLalphaS, CAP1 and Gsalpha expression in AHO-like platelets. A. Immunoblot analysis of XLalphas, CAP1 and Gsalpha protein in platelet lysates from XL hypermethylated AHO-like patients 12, 13, 10, 14, 15, 16 and 3 controls and B. correspondent densitometric scanning of XLalphaS protein in platelet lysates from AHO-like patients with XL hypermethylation (patients 6 to 16) and 5 controls (Controls). Results are expressed as percentage of controls (taken as 100%). Mean as well as SD are depicted as black horizontal and vertical lines, respectively. *, p value<0.05, two-tailed unpaired T-test.

To evaluate whether the XL hypermethylation would be associated with decreased XLalphaS expression, immunoblot analysis was performed using platelet extracts as we previously also did for a PHP-Ib patient with XL hypomethylation and increased XLalphaS levels in platelets [41]. We have studied XLalphaS and Gsalpha expression in platelets from 11 of the 17 patients and 5 healthy controls (**Figure** **3**). While Gsalpha was not statistically different between patients and controls, XLalphaS showed a significant decreased expression (58±32 vs. 100±19, respectively. T-test, p<0.05).

### Study of IGF2 and H19 ICR1 methylation

We next studied 30 CpGs in the DMR1 of *IGF2* and 25 CpGs in the ICR1 of the *H19* locus (**Table** **3** for their precise chromosomal location). Surprisingly, we could observe significant hypermethylation of the *IGF2* amplicon in patients *vs*. controls (20±10 vs. 14±7%; T-test, p<0.05; **Figure** **1B**). The overall CpG methylation for the *H19* amplicon was not significantly different for patients and controls (35±8 vs. 35±5%), though a significant overall hypermethylation was observed for patients 2 and 4 (**Figure** **2B**). For the methylation analysis of single CpGs within the *IGF2* amplicon, we could observe a significant hypermethylation in 14 out of 17 PPHP patients at specific CpGs (**Figure** **2B** **)** (Z-test, p<0.05). For the *H19* region also some specific CpGs show significant differences in methylation but only for a few patients and clustering within patients seemed not be present. Spearman correlation between IGF2 methylation and height of patients was not significant.

### Study of SNURF methylation

The amplicon for *SNURF* included 18 CpGs and a significant hypomethylation in the *SNURF* amplicon was found for patients *vs.* controls (23±6 vs. 32±6%; T-test, p<0.001; **Figure 1C**). Remarkably, single CpG analysis showed both significant hyper (CpG7_8) and hypo (CpG14_16, CpG25) methylation (**Figure** **2C**) within the same amplicon and for almost all patients. This dual pattern was not observed in any of the normal control subjects. Spearman correlation between SNURF methylation and weight of patients was not significant.

### Study of GRB10 methylation

The amplicon for the GRB10 region included 18 CpGs and their methylation did not appear to be significantly different between patients and controls (37±7 vs. 34±6%; **Figure** **1C**). Interestingly, the overall methylation for patients 1 and 2 showed a significant GRB10 hypermethylation of 56 and 50%, respectively, *vs.* 35±6% for controls (Z-test, p<0.05) (**Figure** **2C** **)**. The analysis of single CpGs showed some significant differences for some patients with both hyper- and hypomethylated sites (**Figure** **2C**).

## Discussion

The human *GNAS* cluster contains three differentially methylated regions: NESP, XL and exon A/B [19]. Patients who develop PHP-Ib usually present with exon A/B hypomethylation [14], [45]–[47]. In these familial PHP-Ib cases the latter appears to be caused by maternally inherited deletions affecting either the STX16 [39], [48] or the NESP55/NESPAS regions [40], [49], [50]. Broader *GNAS* imprinting defects involving the three differentially methylated *GNAS* regions are always observed in sporadic PHP-Ib cases with NESP55 hypermethylation versus XL and exon A/B hypomethylation [19], [46], [51]–[53]. Recently, a similar broad epigenetic *GNAS* defect was described for some PHP-Ia cases without *GNAS* coding mutations [15], [16], [18], [19]. These patients had PTH resistance but also an AHO phenotype implicating that *GNAS* methylation defects could also result in AHO features. We therefore hypothesize that patients with an AHO-like phenotype but no endocrine abnormalities and still having functional Gs hypofunction (often referred to as PPHP) could present with GNAS methylation abnormalities if coding *GNAS* mutations are also excluded. We studied *GNAS* methylation in 16 patients with clinical diagnosis of PPHP and 1 POH patient without *GNAS* mutations but having platelet Gs hypofunction and an AHO phenotype that mainly involves short stature and brachydactyly and/or other types of bone abnormalities. *GNAS* methylation was quantified for the three differentially methylated regions using the Sequenom EpiTYPER as we previously did for PHP-Ib and PHP-Ia cases [19]. Grouped analysis showed a significant hypermethylation for the XL amplicon in PPHP patients versus controls (36% vs 29%; p<0.001) but overall methylation for the NESP and ExonA/B regions was not significantly different between patients and controls, except for significant hypermethylation in patients 1, 2 and 3 for NESP and patients 3 and 5 for ExonA/B. The same trend for hypermethylation in NESP and ExonA/B is also visible when analyzing separate CpGs for at least the first 10 patients while the other 7 patients show a weak trend towards hypomethylation of NESP and ExonA/B. This peculiar methylation pattern (with hypermethylation of NESP, XL and ExonA/B) is different from the imprinting pattern observed in PHP-Ib and PHP-Ia patients (having NESP hyper versus XL and Exon A/B hypomethylation).

The main defect in our patients is the significant XL hypermethylation that could be linked to their Short for Gestational Age (SGA) and shortness phenotype. Interestingly, it is known that the main phenotype for XLalphaS deficient mice is the regulation of postnatal growth with neonatal feeding problems, leanness, inertiae and a high mortality rate [54]. Postnatally, changes in the expression pattern of XLalphaS in different tissues have been also characterized, as surviving mice develop into healthy and fertile adults, which are however characterized by leanness despite elevated food intake [55]. In addition, GNAS deletions including the XL region have been identified in some patients with severe pre- and/or postnatal growth retardation as well as feeding difficulties [56], [57]. We also found that the XL hypermethylation in the patients was associated with decreased XLalphaS protein levels in their platelets. Further studies will be needed to evaluate whether this decreased expression of XLalphaS could also be responsible for the platelets Gs hypofunction in these patients. We have previously shown that XLalphas can regulate platelet Gs activity [43], [58], data that have been further supported by studies in other cells [59]–[61].

Some typical AHO features are also present in patients with other imprinting syndromes such as for the growth and neurodevelopmental diseases Silver-Russel, Beckwith-Wiedemann, Prader-Willi and Angelman syndromes. In addition, *IGF2, H19* and GRB10 together with *GNAS* have been described to be part of an imprinted gene network that regulate embryonic growth and differentiation dependent on Zac-1 regulation in mice [27]. Therefore, we have also studied the methylation of other imprinted genes such as *IGF2*, *H19*, *SNURF* and GRB10. Surprisingly, we could observe significant hypermethylation for *IGF2* (20 *vs*. 10%; P<0.05) and hypomethylation (23 *vs*. 32; P<0.001) for *SNURF* while *H19* and GRB10 showed no overall differences between patients and controls. The physiological relevance of these findings in relation to the clinical phenotypes remains to be studied. However, some other groups already reported so-called multilocus methylation abnormalities (e.g. for Beckwith-Wiedemann syndrome [62] and Silver-Russel syndrome [63], [64]). In all these reports somatic mosaicism has been proposed to explain the patients epigenotypes as result of a post-zygotic error of imprint setting. Interestingly, a similar overall methylation defect has been recently described in patients with growth and development problems [65]–[67].

Mutations in a trans-acting factor involved in establishing or maintaining methylation at multiple chromosomal loci however could also explain the presence of such overall methylation abnormalities. The latter hypothesis has been demonstrated in the Beckwith-Wiedemann syndrome [68], transient neonatal diabetes [69] and the Immunodeficiency-Centromeric instability-Facial anomalies (ICF) syndrome [70]. A similar mechanism has also been recently postulated to exists for PHPIb cases [71] but this remain to be proven. The methylation changes observed in our patients seem to affect mainly maternally methylated regions as XL, IGF2 and SNURF are paternally expressed genes (see **Figures S1, S2, S3**). In conclusion we studied *GNAS*, *IGF2*, *H19*, *SNURF* and *GRB10* methylation in patients with and AHO-like phenotype and Gs hypofunction but no *GNAS* coding mutations. We could broaden the spectrum of (epi)genetic defects associated with an AHO phenotype by identifying an epigenetic defect in XL, *IGF2* and *SNURF* in 16 PPHP patients and 1 POH case. More studies on multiple imprinting control regions in more PPHP patients are warranted to further investigate the combination of epigenetic defects in relation to phenotypes.

## Supporting Information

Figure S1 **GNAS schematic representation of genomic regions studied via Sequenom EpiTYPER.** GNAS schematic representation of genomic regions studied via Sequenom EpiTYPER. Features of the paternal and the maternal allele are shown above and below the line, respectively. The arrows show initiation and direction of transcription. Paternal and maternal transcripts are highlighted in blue and pink, respectively. The first exons of the protein coding transcripts are shown as black boxes and the first exons of the noncoding transcripts (Nespas and exon A/B) are shown as gray boxes. Differentially methylated regions (DMRs) are shown by + symbols (indication of methylation). For each amplicon reported in the black frames CpG sites are underlined, CpGs studied via Sequenom are additionally depicted in italic and bold. Red dinucleotides refer to SNPs analysed in the same regions. The figure is not to scale. Adapted from Izzi et al. Curr Mol Med 2012.
(TIF)

Figure S2 **IGF2/H19 schematic representation of genomic regions studied via Sequenom EpiTYPER.** Features of the paternal and the maternal allele are shown above and below the line, respectively. The arrows show initiation and direction of transcription. Paternal IGF2 transcript is highlighted in blue. The first exons of the protein coding transcripts are shown as black boxes. Differentially methylated regions (DMRs) are shown by + symbols (indication of methylation). For each amplicon reported in the black frames CpG sites are underlined, CpGs studied via Sequenom are additionally depicted in italic and bold. Red dinucleotides refer to SNPs analysed in the same regions. The figure is not to scale. Adapted from Jeong et al. Nature Genetics (2004) **36**, 1036–1037.
(TIF)

Figure S3 **SNURF (A) and GRB10 (B) schematic representation of genomic regions studied via Sequenom EpiTYPER.** Features of the paternal and the maternal allele are shown above and below the line, respectively. The arrows show initiation and direction of transcription. Paternal SNURF transcript is highlighted in blue. The first exons of the protein coding transcripts are shown as black boxes. Differentially methylated regions (DMRs) are shown by + symbols (indication of methylation). For each amplicon reported in the black frames CpG sites are underlined, CpGs studied via Sequenom are additionally depicted in italic and bold. Red dinucleotides refer to SNPs analysed in the same regions. The figure is not to scale. **B** adapted from Hikichi et al. Nucleic Acids Research (2003) 31 (5): 1398–1406.
(TIF)

Table S1 (XLSX)

Table S2 (XLSX)

Table S3 (XLSX)
