# Supplementary figures and images for: Methylation Defect in Imprinted Genes Detected in Patients with an Albright's Hereditary Osteodystrophy Like Phenotype and Platelet Gs Hypofunction
Source: PLoS One. 2012 Jun 5;7(6):e38579. doi: 10.1371/journal.pone.0038579 (PMC3367970; doi:10.1371/journal.pone.0038579)

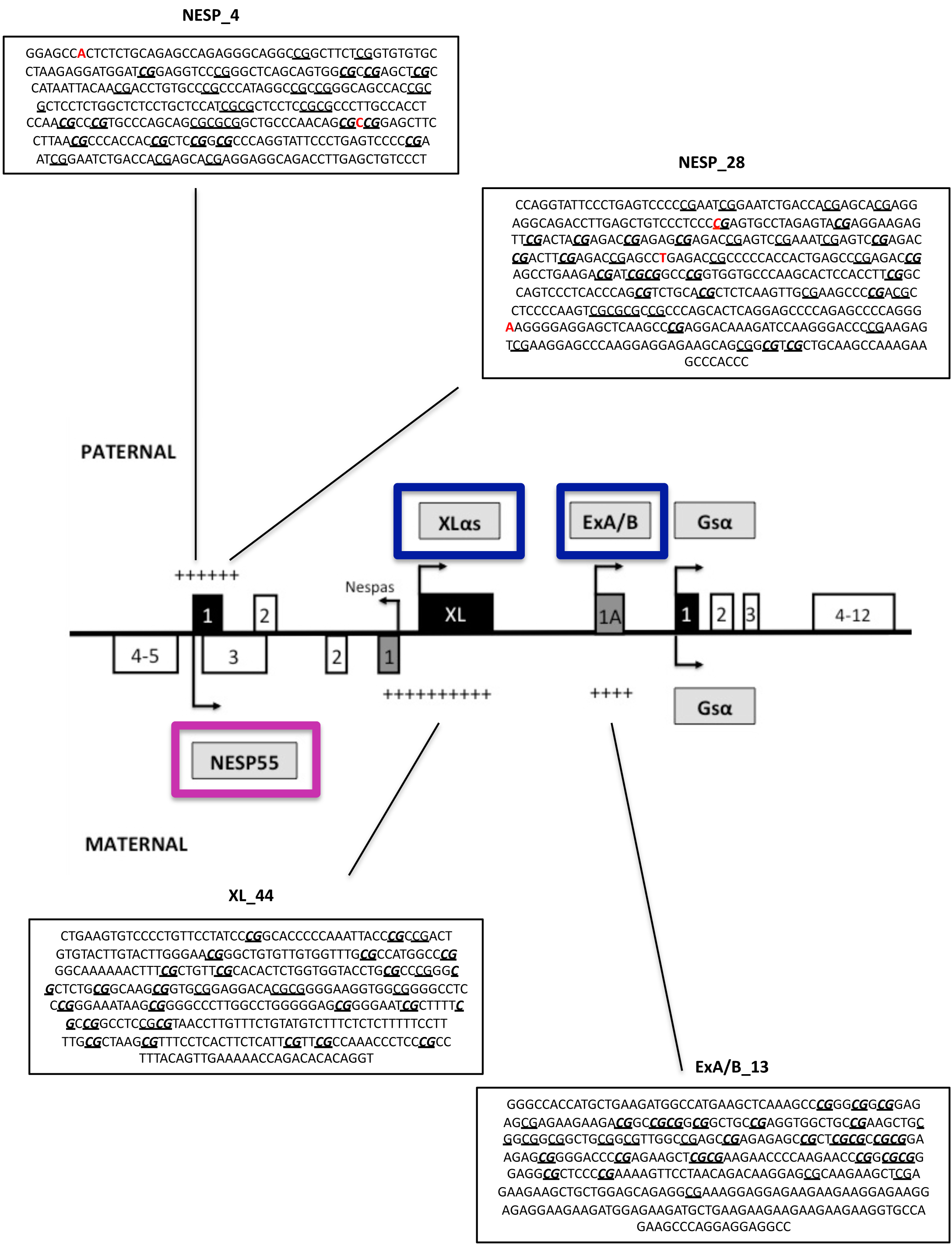

Supplement: Figure S1 — GNAS schematic representation of genomic regions studied via Sequenom EpiTYPER. GNAS schematic representation of genomic regions studied via Sequenom EpiTYPER. Features of the paternal and the maternal allele are shown above and below the line, respectively. The arrows show initiation and direction of transcription. Paternal and maternal transcripts are highlighted in blue and pink, respectively. The first exons of the protein coding transcripts are shown as black boxes and the first exons of the noncoding transcripts (Nespas and exon A/B) are shown as gray boxes. Differentially methylated regions (DMRs) are shown by + symbols (indication of methylation). For each amplicon reported in the black frames CpG sites are underlined, CpGs studied via Sequenom are additionally depicted in italic and bold. Red dinucleotides refer to SNPs analysed in the same regions. The figure is not to scale. Adapted from Izzi et al. Curr Mol Med 2012. (TIF) [file pone.0038579.s001.tif]

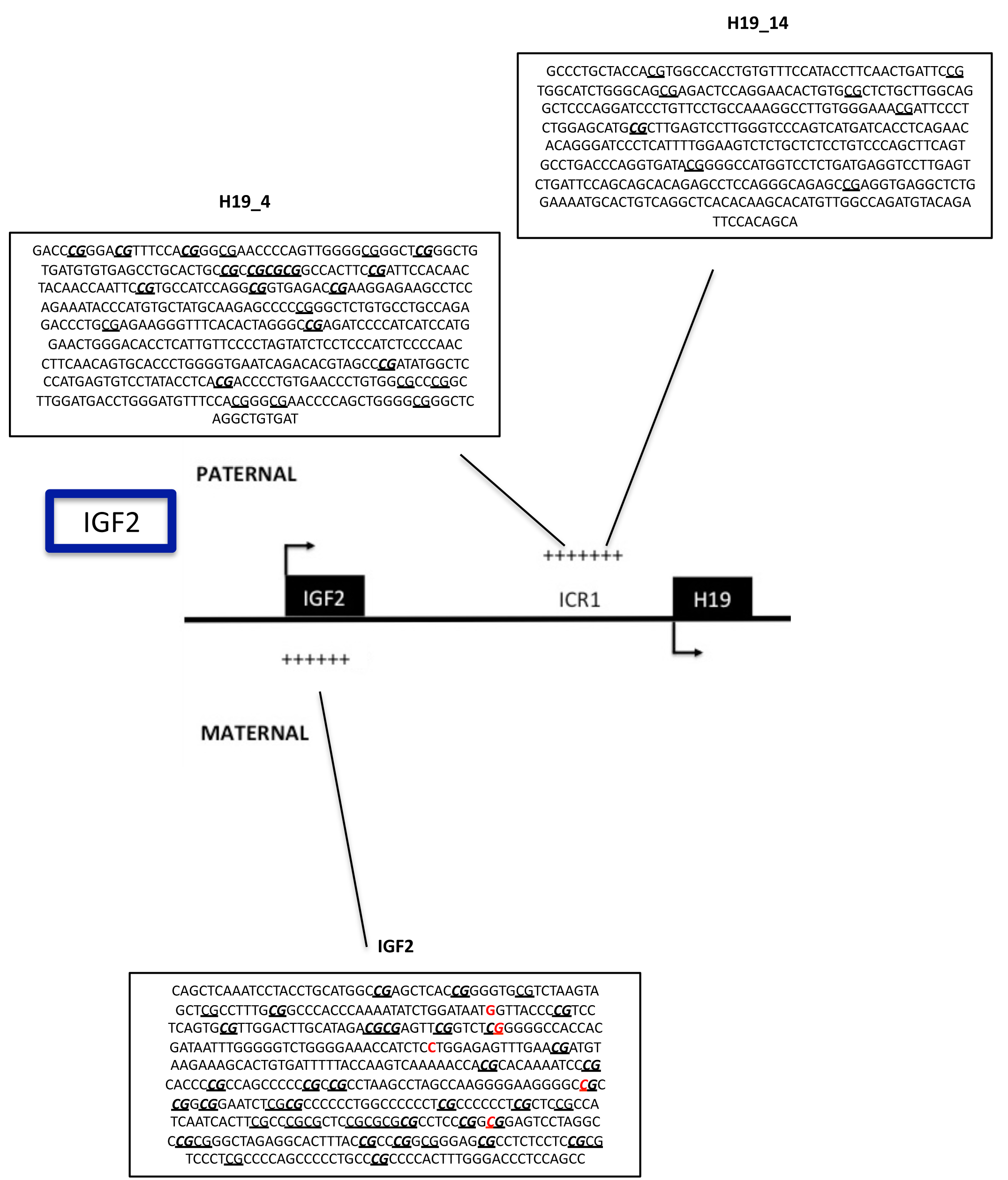

Supplement: Figure S2 — IGF2/H19 schematic representation of genomic regions studied via Sequenom EpiTYPER. Features of the paternal and the maternal allele are shown above and below the line, respectively. The arrows show initiation and direction of transcription. Paternal IGF2 transcript is highlighted in blue. The first exons of the protein coding transcripts are shown as black boxes. Differentially methylated regions (DMRs) are shown by + symbols (indication of methylation). For each amplicon reported in the black frames CpG sites are underlined, CpGs studied via Sequenom are additionally depicted in italic and bold. Red dinucleotides refer to SNPs analysed in the same regions. The figure is not to scale. Adapted from Jeong et al. Nature Genetics (2004) 36, 1036–1037. (TIF) [file pone.0038579.s002.tif]

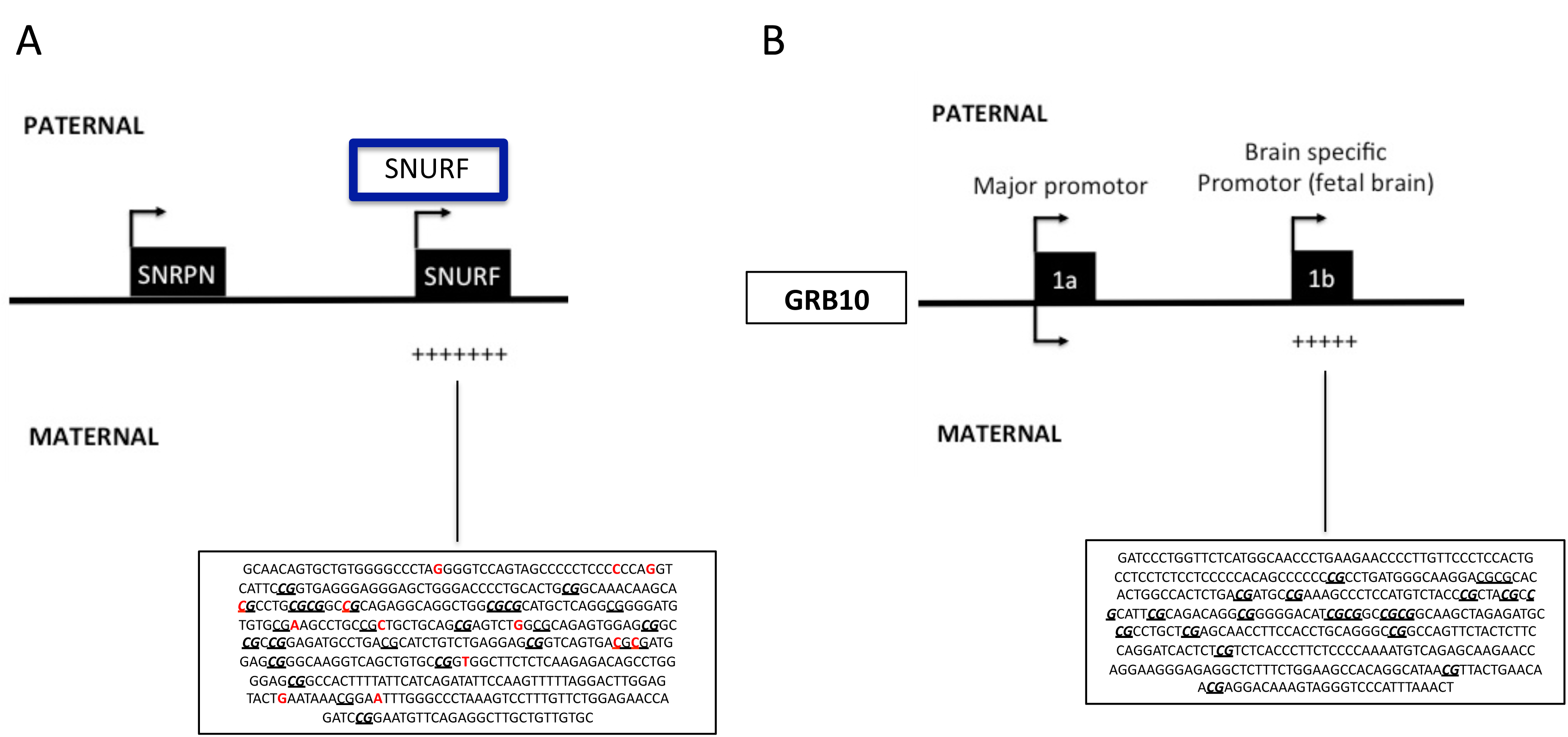

Supplement: Figure S3 — SNURF (A) and GRB10 (B) schematic representation of genomic regions studied via Sequenom EpiTYPER. Features of the paternal and the maternal allele are shown above and below the line, respectively. The arrows show initiation and direction of transcription. Paternal SNURF transcript is highlighted in blue. The first exons of the protein coding transcripts are shown as black boxes. Differentially methylated regions (DMRs) are shown by + symbols (indication of methylation). For each amplicon reported in the black frames CpG sites are underlined, CpGs studied via Sequenom are additionally depicted in italic and bold. Red dinucleotides refer to SNPs analysed in the same regions. The figure is not to scale. B adapted from Hikichi et al. Nucleic Acids Research (2003) 31 (5): 1398–1406. (TIF) [file pone.0038579.s003.tif]
